# Skip N2 Metastasis in Resected Lung Cancer: Prognostic Subclassification by Lymphatic Drainage and Histology

**DOI:** 10.5761/atcs.oa.26-00068

**Published:** 2026-08-22

**Authors:** Shixiong Yao, Chun Zheng, Chaobin Zhang

**Affiliations:** 1 Department of Cardiothoracic Surgery, Xiaogan First People’s Hospital Thoracic Surgery, Xiaogan, Hubei, China; 2 Department of Cardiovascular Medicine, Xiaogan First People’s Hospital Thoracic Surgery, Xiaogan, Hubei, China

**Keywords:** non-small-cell lung cancer, skip N2 metastasis, lymphatic drainage, prognostic classification, histological characteristics

## Abstract

**Purpose:**

Skip N2 metastasis in non-small-cell lung cancer (NSCLC) confers a favorable prognosis, yet the biological determinants remain poorly defined. We investigated lymphatic drainage patterns and histological characteristics to develop a novel pN2 subclassification.

**Methods:**

We retrospectively analyzed 180 consecutive pN2 patients (2015–2022); 50 exhibited skip metastasis. Lymphatic drainage was classified as direct, indirect, or mixed, and a composite score incorporating drainage, histology, grade, and lymphovascular invasion stratified patients into 3 risk groups. Survival was assessed with Kaplan–Meier and Cox models.

**Results:**

Skip metastasis was associated with superior KM-estimated 5-year overall survival compared to non-skip disease (56% vs 41%; median follow-up 43 months, with 34% of patients reaching the 5-year assessment). Direct drainage predominated in peripheral upper-lobe tumors and correlated with lower lymphovascular invasion. The model distinguished 3 prognostic groups: low-risk (5-year survival 71%), intermediate-risk (55%), and high-risk (27%). The subclassification was an independent prognostic factor, with high-risk patients showing an adjusted hazard ratio of 2.45 (95% CI 1.42–4.23).

**Conclusion:**

Integrating lymphatic drainage and histology refines prognosis in pN2 lung cancer and provides a hypothesis-generating framework for prognostic stratification of skip N2 disease, with potential to inform future clinical trial design pending validation.

## Introduction

Non-small-cell lung cancer (NSCLC) accounts for approximately 85% of all lung cancers, and patients with mediastinal lymph-node metastasis (pN2) continue to face 5-year survival rates of only 20%–40% even after complete resection and adjuvant chemotherapy.^1,2)^ The eighth edition TNM classification regards all pN2 disease as a single entity, yet accumulating data show wide biological heterogeneity within this stage, underscoring the need for finer prognostic tools that can separate patients who require aggressive multimodal therapy from those who might safely be spared excessive treatment.^3)^

One of the most clinically relevant, but still underutilized, observations within pN2 disease is the “skip metastasis” phenomenon, defined as the presence of tumor deposits in mediastinal nodes (N2) without identifiable involvement of hilar or peribronchial nodes (N1).^4)^ Population-based series report skip metastasis in 17%–42% of resected pN2 cases, and multiple cohort studies have consistently associated this pattern with a 10%–15% absolute improvement in 5-year overall survival.^5,6)^ These findings challenge the traditional stepwise lymphatic spread model and suggest that skip metastasis may reflect distinct anatomical drainage patterns rather than a simple staging curiosity.^7)^ Despite this, current guidelines make no provision for incorporating skip status into treatment algorithms, and the factors that drive the favorable prognosis remain poorly characterized.^8)^

We therefore undertook the present study to develop a practical, biologically grounded subclassification for resected pN2 NSCLC. By integrating the mechanism of lymphatic drainage with routinely reported histological variables, we constructed a 3-tier risk model and validated its ability to predict long-term survival in a single-institutional retrospective cohort. Our goal was to provide clinicians with a simple, reproducible tool that refines prognostic counselling in pN2 NSCLC. While we discuss potential implications for future trial design, we emphasize that the present study does not provide evidence sufficient to guide individualized postoperative adjuvant treatment. We acknowledge that a newer N2 subclassification (N2a/N2b) has been proposed based on nodal station involvement; however, the present study focuses on biological heterogeneity within skip N2 disease rather than anatomical staging refinements.

## Materials and Methods

### Study design and patient selection

This retrospective cohort study was conducted at a single high-volume thoracic surgery center and included consecutive patients who underwent curative-intent surgical resection for NSCLC between January 2015 and December 2022. The study protocol was approved by the Institutional Review Board (Xiaoyiyilunshen [05] -05) and complied with the Declaration of Helsinki. Informed consent was waived due to the retrospective nature of the study. All patient data were de-identified to ensure confidentiality.

Patients meeting the following criteria were included: (1) age ≥18 years, (2) pathologically confirmed NSCLC according to the 2021 WHO classification of thoracic tumors,^9)^ (3) pathological stage pN2 based on the 8th edition TNM staging system, (4) complete resection (R0) with systematic mediastinal lymph node dissection, and (5) availability of complete follow-up data. Exclusion criteria comprised small cell lung carcinoma or other neuroendocrine tumors, sublobar resections, preoperative neoadjuvant therapy, incomplete lymph node evaluation, and synchronous malignancies.

### Pathological evaluation and definition of skip N2 metastasis

All surgical specimens were fixed in 10% neutral-buffered formalin and processed according to standard protocols. Hematoxylin and eosin staining was performed on 4-µm sections for histopathological assessment. Skip N2 metastasis was strictly defined as the presence of metastasis in mediastinal lymph nodes (N2 stations) in the absence of any involvement of intrapulmonary lymph nodes (N1 stations). Lymph node mapping followed the International Association for the Study of Lung Cancer (IASLC) nodal chart, with N1 stations comprising hilar, interlobar, lobar, and segmental nodes (stations 10–12), and N2 stations including upper, aortic, and lower mediastinal nodes (stations 1–9). All lymph node stations were pathologically examined, and the diagnosis of skip N2 metastasis was independently confirmed by 2 thoracic pathologists with >10 years of experience; discrepancies were resolved through consensus review or consultation with a third senior pathologist. Histological classification was performed according to the 2021 WHO criteria,^9)^ categorizing tumors as adenocarcinoma (including predominant subtypes: lepidic, acinar, papillary, micropapillary, and solid), squamous cell carcinoma, or other rare types (adenosquamous, sarcomatoid carcinoma). Histological grade was assessed as low (G1), intermediate (G2), or high (G3) based on nuclear atypia and mitotic activity. Lymphovascular invasion (LVI) was identified immunohistochemically using D2-40/podoplanin staining, while vascular invasion was assessed with CD31/CD34 immunostaining. Pleural invasion was classified according to the degree of visceral pleural involvement (PL0 to PL3). Tumor necrosis was semi-quantitatively graded as absent (<10%), moderate (10%–50%), or extensive (>50%).

### Classification of lymphatic drainage patterns

Lymphatic drainage patterns were retrospectively classified through integrated evaluation of tumor location, radiological features, and pathological lymph node mapping. Classification was performed independently by 2 thoracic surgeons with >10 years of experience; discrepancies were resolved by consensus with a third senior surgeon. We explicitly acknowledge that the proposed “direct” and “indirect” drainage patterns are inferred from anatomical surrogates (tumor peripherality/centrality and nodal station distribution) rather than directly validated biological pathways. The criteria for each pattern were predefined as follows:

Direct drainage pattern was defined when all 3 of the following conditions were met: (i) the tumor was located in the outer third of the lung parenchyma (peripheral location); (ii) the tumor was in the upper lobe or lower lobe with anatomically established subpleural lymphatic channels to specific mediastinal stations (right upper lobe → stations 2R/4R; left upper lobe → stations 5/6^10)^; lower lobes → stations 7/8/9); and (iii) skip N2 metastasis was present exclusively in the mediastinal stations anatomically corresponding to the tumor lobe, without any N1 station involvement.

Indirect drainage pattern was defined when: (i) the tumor was located in the inner or middle third of the lung parenchyma (central or mid-zone location); or (ii) the tumor was peripheral but with skip N2 metastasis in mediastinal stations not anatomically corresponding to the established subpleural drainage routes of the primary lobe; or (iii) there was presence of any suspicious radiological or pathological features suggestive of an occult N1 micrometastatic burden (e.g., lymphovascular invasion, peribronchial soft tissue thickening on computed tomography (CT) without definitive nodal enlargement). This peripheral location criterion is grounded in anatomical studies demonstrating that subpleural lymphatic plexuses in peripheral lung zones drain directly to mediastinal nodes.^11,12)^

Mixed pattern included cases with features of both direct and indirect drainage (e.g., a peripheral upper-lobe tumor with both ipsilateral paratracheal and contralateral mediastinal nodal involvement), or atypical drainage routes not conforming to established anatomical pathways. Interobserver agreement for drainage pattern classification was assessed in a random subset of 20 cases (40.0%) reviewed independently by 2 thoracic surgeons; Cohen’s kappa coefficient was 0.82 (95% confidence interval [CI] 0.71–0.93), indicating substantial agreement.

### Radiological assessment

All patients underwent contrast-enhanced chest CT within 4 weeks preoperatively. Tumor size was measured as the maximal diameter on CT images. Tumor location was recorded as right upper lobe, right middle lobe, right lower lobe, left upper lobe, or left lower lobe. Tumor centrality was defined based on distance from the hilum, with peripheral tumors located in the outer third of the lung parenchyma. Positron emission tomography (PET-CT) data, when available, were reviewed to record the maximum standardized uptake value (SUVmax) of the primary tumor and of each pathologically positive lymph node station.

### Data collection

Clinical data extracted from electronic medical records included patient demographics (age, gender, smoking history in pack-years), Eastern Cooperative Oncology Group (ECOG) performance status, and Charlson Comorbidity Index (CCI). Surgical details comprised operative approach (thoracotomy vs minimally invasive), type of resection (lobectomy, bilobectomy, pneumonectomy), and number of lymph node stations dissected. Pathological data included T category, histological type and grade, presence of LVI, vascular invasion, pleural invasion, and total number of lymph nodes harvested. Adjuvant treatment information was documented, including chemotherapy regimens and completion status. Follow-up data were extracted from scheduled hospital visits and, when necessary, from structured telephone interviews approved by the institutional review board. Surveillance adhered to the institutional protocol: chest CT and clinical review every 6 months for the first 2 years, every 6–12 months for years 3–5, and annually thereafter.

### Study endpoints

The primary endpoint was overall survival (OS), defined as the interval from the date of surgery to the date of death from any cause or last follow-up. Secondary endpoints included disease-free survival, calculated from surgery to the first documented recurrence (local or distant) or death, and lung cancer-specific survival. Recurrence patterns were classified as local–regional (involving the ipsilateral hemithorax) or distant metastasis.

### Statistical analysis

Statistical analyses were performed using R software (version 4.2.1). Continuous variables were presented as mean ± standard deviation or median with interquartile range, as appropriate, and compared using Student’s t-test or the Mann-Whitney U test. Categorical variables were expressed as frequencies and percentages, with comparisons made using the chi-square test or Fisher’s exact test. Five-year survival probabilities were estimated by the Kaplan–Meier method, with 95% CIs calculated via log–log transformation. Univariate and multivariate Cox proportional hazards regression models were employed to identify prognostic factors, with results expressed as hazard ratios (HRs) and 95% CIs. Variables with p <0.10 in univariate analysis were entered into the multivariate model using a stepwise backward elimination approach.

A novel prognostic subclassification system was developed by integrating lymphatic drainage patterns and histological characteristics. Each patient received a composite risk score based on the following criteria: direct drainage pattern (0 points), indirect drainage pattern (1 point), or mixed pattern (2 points); adenocarcinoma histology (0 points), squamous cell carcinoma (1 point), or other types (2 points); low-intermediate grade (G1–2) (0 points) vs high grade (G3) (1 point); and absence (0 points) vs presence (1 point) of LVI. Patients were stratified into 3 risk groups: low-risk skip N2 (score 0–1), intermediate-risk skip N2 (score 2–3), and high-risk skip N2 (score ≥4). We acknowledge that variables incorporated into the scoring system (grade, lymphovascular invasion) were also included in multivariate analysis, which may raise concerns regarding circularity. However, the score was constructed a priori based on anatomical and histological patterns observed in clinical practice rather than data-driven optimization, and the multivariate model tested the composite score as a single variable rather than its individual components, mitigating overfitting risk. The discriminatory ability of this classification was evaluated using Harrell’s concordance index (C-index) and compared with conventional N2 staging using time-dependent ROC curve analysis. All statistical tests were 2-sided, with a significance level of p <0.05.

## Results

### Patient enrollment and baseline characteristics

**Fig. 1:**
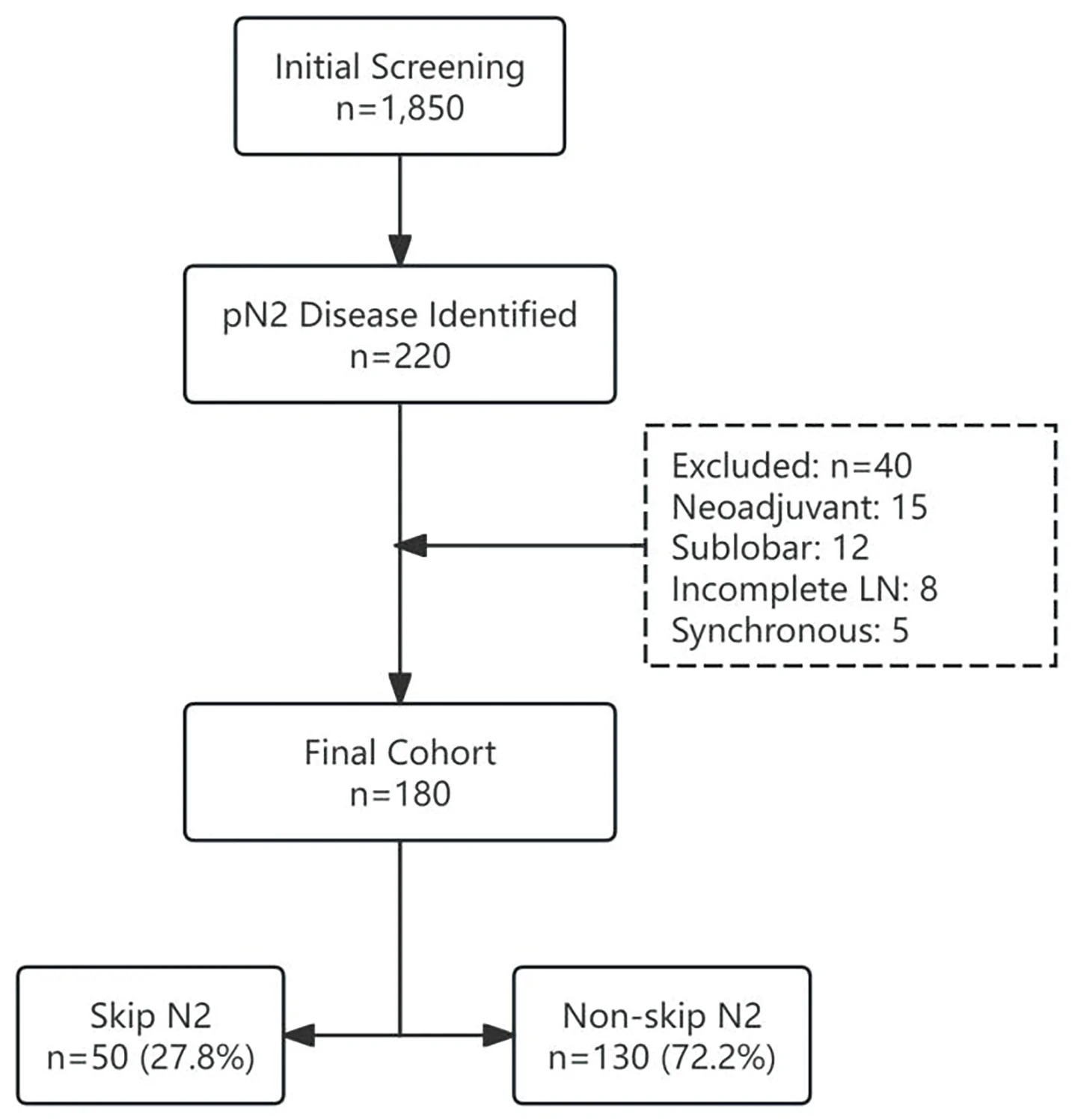
Patient enrollment flow diagram. The flowchart depicts patient selection from the initial surgical cohort (n = 1850) through screening and exclusion to the final analysis groups (skip N2 n = 50, non-skip N2 n = 130).

Between January 2015 and December 2022, 1850 patients underwent surgical resection for NSCLC, of whom 220 had pathologically confirmed pN2 disease. After excluding 40 patients who received neoadjuvant therapy, underwent sublobar resection, had incomplete lymph node evaluation, or harbored synchronous malignancies, the final cohort comprised 180 patients, including 50 (27.8%) with skip N2 metastasis and 130 (72.2%) with non-skip N2 metastasis (**Fig. 1**).

Median follow-up was 43 months (range 3–87), with a 92.8% follow-up rate. Skip N2 patients were significantly younger (median age 61 vs 66 years, p = 0.021) and had a lower comorbidity burden (median CCI 2 vs 3, p = 0.034). They exhibited more favorable tumor biology, with smaller median tumor size (3.1 vs 3.7 cm, p <0.001), higher frequency of adenocarcinoma (70.0% vs 56.9%, p = 0.047), and lower rates of high-grade histology (34.0% vs 52.3%, p = 0.018). Skip N2 tumors were more commonly located in upper lobes (62.0% vs 46.2%, p = 0.034) and peripheral locations (56.0% vs 30.8%, p <0.001), with significantly reduced lymphovascular invasion (36.0% vs 59.2%, p = 0.003), vascular invasion (20.0% vs 39.2%, p = 0.011), and pleural invasion (30.0% vs 45.4%, p = 0.045). No significant differences were observed in gender distribution, smoking prevalence, ECOG performance status, surgical approach, or adjuvant chemotherapy receipt between groups (**Table 1**).

**Table 1:** Baseline clinicopathological characteristics of study cohort (n = 180)

Variable	Skip N2 (n = 50)	Non-skip N2 (n = 130)	p-Value
Demographics			
Age, median (range)	61 (45–78)	66 (48–82)	0.021
Male gender, n (%)	32 (64.0)	78 (60.0)	0.612
Smoking history (pack-years), median	30 (0–80)	38 (0–110)	0.012
Ever-smoker, n (%)	38 (76.0)	95 (73.1)	0.688
ECOG PS 0–1, n (%)	46 (92.0)	116 (89.2)	0.587
Charlson Comorbidity Index, median	2 (0–5)	3 (0–7)	0.034
Tumor characteristics			
Tumor size (cm), median	3.1 (1.5–5.5)	3.7 (1.8–6.2)	<0.001
Upper-lobe location, n (%)	31 (62.0)	60 (46.2)	0.034
Peripheral location, n (%)	28 (56.0)	40 (30.8)	<0.001
T category, n (%)			0.029
T1–T2	38 (76.0)	78 (60.0)	
T3–T4	12 (24.0)	52 (40.0)	
Histology			
Adenocarcinoma, n (%)	35 (70.0)	74 (56.9)	0.047
Squamous cell carcinoma, n (%)	13 (26.0)	49 (37.7)	0.117
Other types, n (%)	2 (4.0)	7 (5.4)	0.711
Histological grade G3, n (%)	17 (34.0)	68 (52.3)	0.018
Pathological features			
Lymphovascular invasion, n (%)	18 (36.0)	77 (59.2)	0.003
Vascular invasion, n (%)	10 (20.0)	51 (39.2)	0.011
Pleural invasion, n (%)	15 (30.0)	59 (45.4)	0.045
Surgical details			
Minimally invasive approach, n (%)	33 (66.0)	81 (62.3)	0.638
Lobectomy, n (%)	42 (84.0)	106 (81.5)	0.688
Lymph node stations dissected, median	5 (3–7)	5 (3–8)	0.845
Total lymph nodes harvested, median	15 (8–28)	18 (9–35)	0.042
Adjuvant treatment			
Adjuvant chemotherapy, n (%)	31 (62.0)	89 (68.5)	0.398
Platinum-based doublet, n (%)	27/31 (87.1)	81/89 (91.0)	0.672

### Lymphatic drainage pattern distribution

**Fig. 2:**
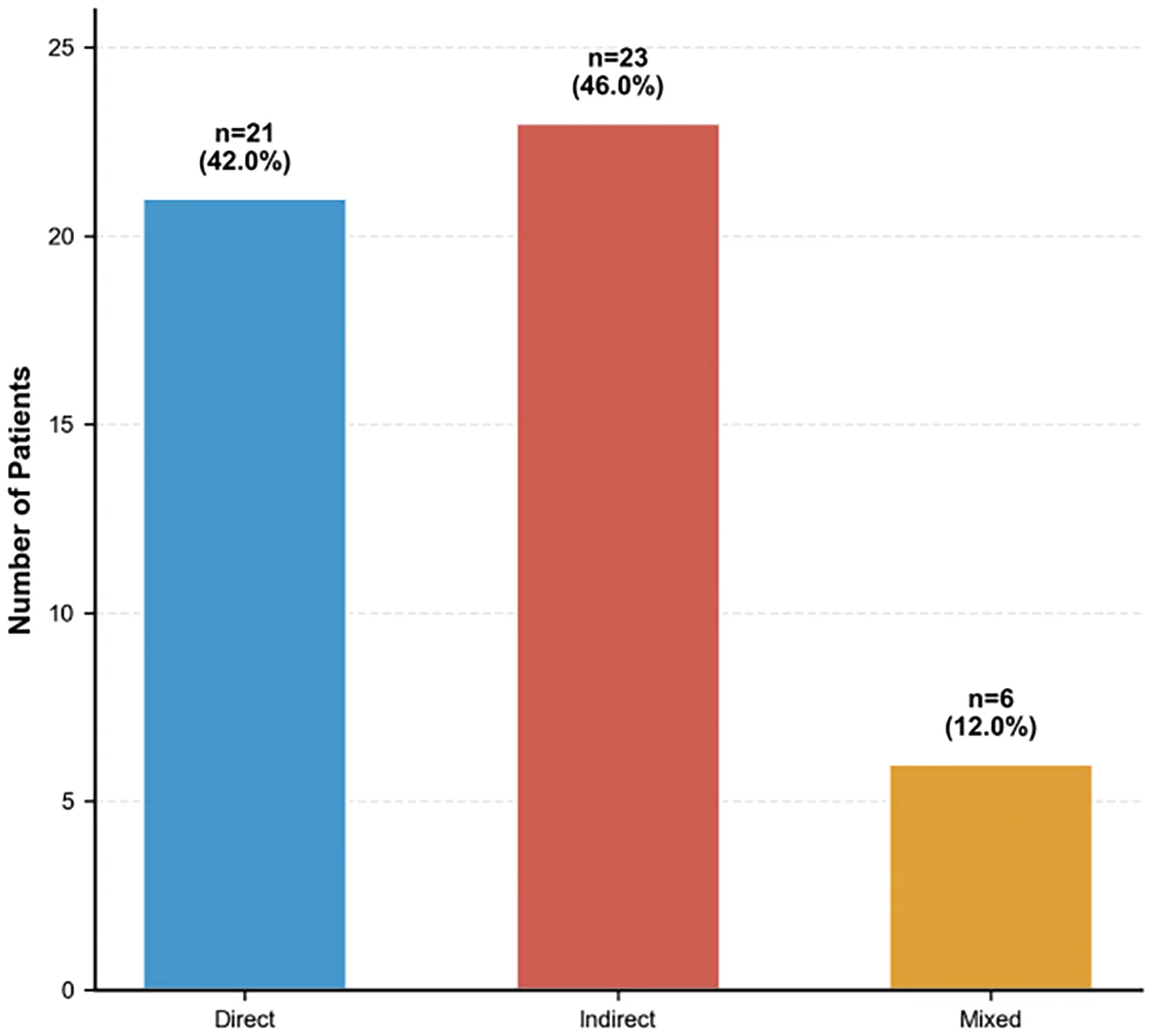
Distribution of lymphatic drainage patterns in skip N2 patients.

Among the 50 skip N2 patients, lymphatic drainage patterns were classified as direct in 21 cases (42.0%), indirect in 23 cases (46.0%), and mixed in 6 cases (12.0%). The direct drainage pattern was predominantly observed in peripheral upper-lobe tumors (76.2% of direct pattern cases), with metastasis most commonly involving paratracheal (stations 2R/4R) or aortopulmonary window stations (stations 5/6). The indirect pattern was more frequent in central tumors and was associated with a higher incidence of lymphovascular invasion (61.0% vs 23.8% in the direct pattern, p = 0.009, **Fig. 2**). Mixed pattern cases demonstrated intermediate characteristics but showed the highest proportion of high-grade histology (50.0%) and solid-predominant adenocarcinoma subtypes (33.3%).

### Survival outcomes

**Fig. 3:**
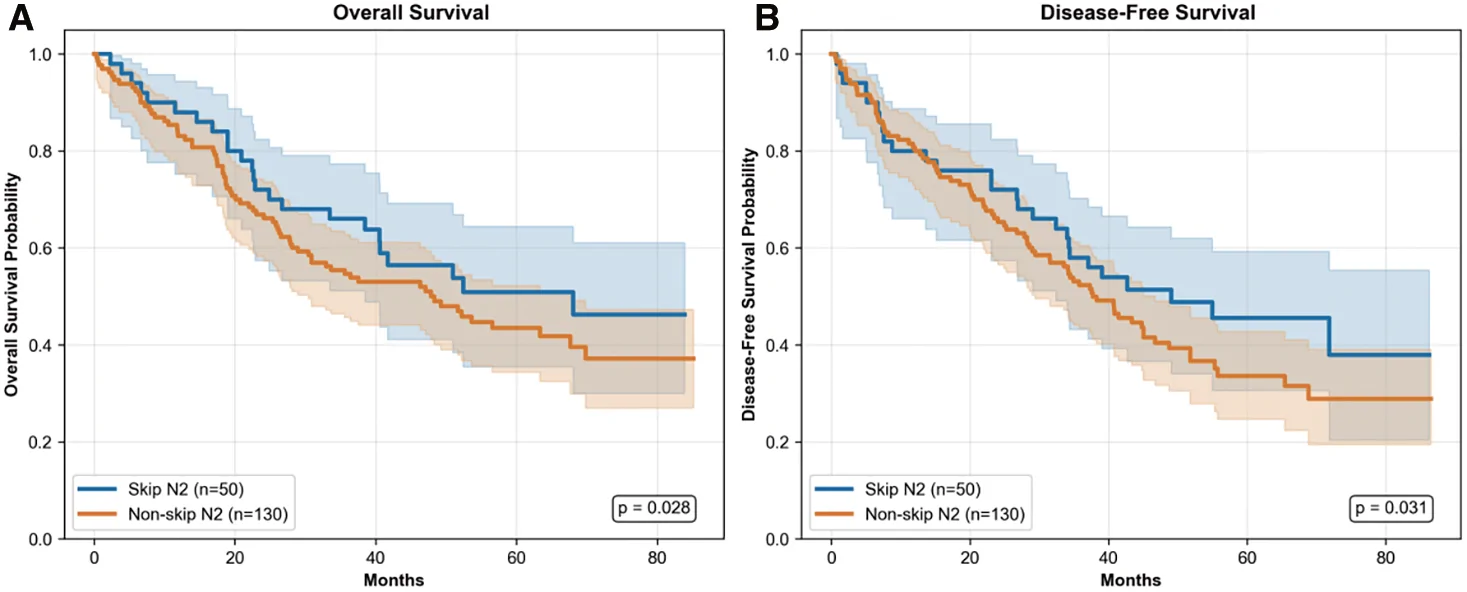
Kaplan–Meier survival curves. (**A**) Overall survival and (**B**) disease-free survival comparing skip N2 versus non-skip N2 patient groups, with numbers at risk indicated below each plot. Five-year survival probabilities are KM-estimated; median follow-up was 43 months, with 34% of patients reaching the 5-year assessment.

Skip N2 metastasis was associated with significantly improved survival outcomes compared to non-skip N2 disease (**Fig. 3**). The KM-estimated 5-year overall survival rate for skip N2 patients was 56.0% (95% CI 43%–69%) versus 41.5% (95% CI 33%–50%) for non-skip N2 patients (p = 0.028; median follow-up 43 months). Median OS was 64 months (95% CI: 52–76) in the skip N2 group versus 48 months (95% CI: 39–57) in the non-skip N2 group. Similarly, the 5-year disease-free survival rate was higher in skip N2 patients (50.0% vs 35.4%, p = 0.031), with median disease-free survival of 52 months (95% CI: 41–63) versus 38 months (95% CI: 30–46).

### Novel prognostic subclassification validation

**Fig. 4:**
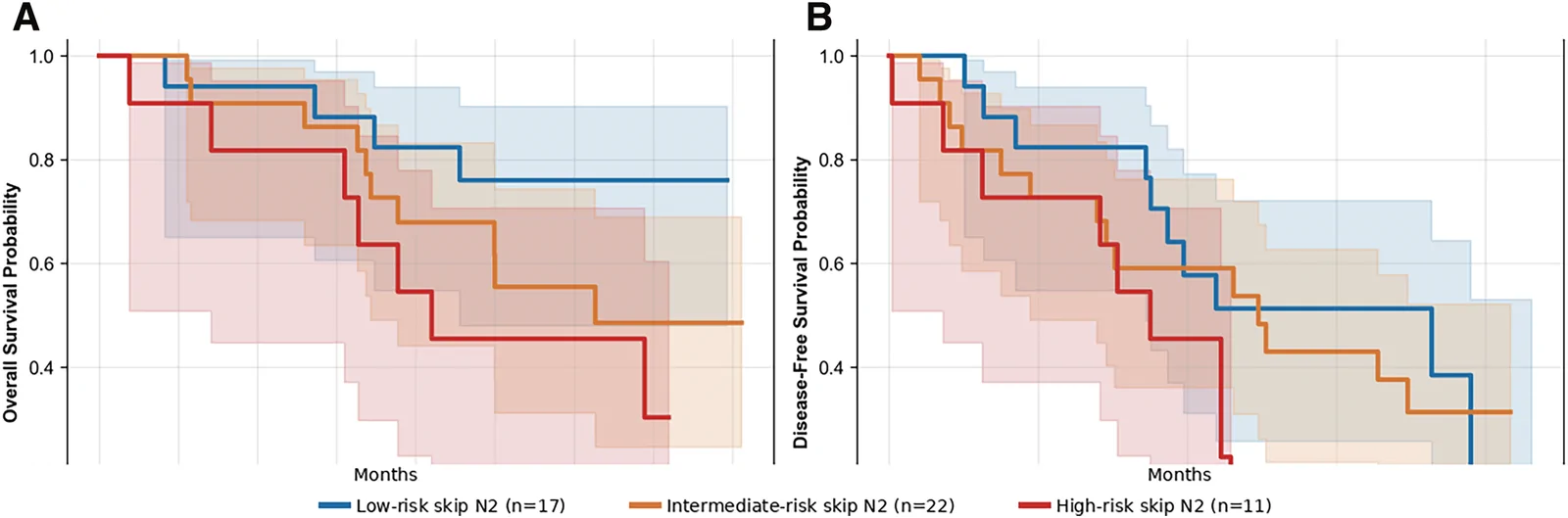
Prognostic subclassification in skip N2 patients. (**A**) Overall survival and (**B**) disease-free survival according to the novel low-risk skip N2, intermediate-risk skip N2, and high-risk skip N2 risk groups, demonstrating incremental prognostic discrimination. KM-estimated 5-year OS rates are shown; median follow-up was 43 months.

Application of our composite risk scoring system to the 50 skip N2 patients stratified them into 3 distinct prognostic groups (**Fig. 4**): low-risk skip N2 (n = 17, 34.0%), intermediate-risk skip N2 (n = 22, 44.0%), and high-risk skip N2 (n = 11, 22.0%). KM-estimated 5-year overall survival rates (median follow-up 43 months) were 70.6% (95% CI 54%–87%) for low-risk skip N2, 54.5% (95% CI 37%–72%) for intermediate-risk skip N2, and 27.3% (95% CI 6%–49%) for high-risk skip N2 (p = 0.001). Median OS values were 78 months, 61 months, and 42 months, for low-risk, intermediate-risk, and high-risk skip N2, respectively. Disease-free survival at 5 years was similarly stratified: 64.7% in low-risk skip N2, 45.5% in intermediate-risk skip N2, and 27.3% in high-risk skip N2 (p = 0.002). The Harrell’s concordance index for the new classification system was 0.67, significantly superior to conventional N2 staging alone (C-index 0.54, p = 0.008). While the score was constructed a priori, the limited sample size (n = 50 skip N2) precludes reliable assessment of model stability, and the C-index of 0.67 may be optimistically biased. To assess internal validity and mitigate overfitting concerns, we performed 1000 bootstrap resamples of the skip N2 cohort. The optimism-corrected C-index was 0.64 (95% CI 0.58–0.70), confirming reasonable discriminatory stability despite the limited sample size.

### Multivariate prognostic analysis

Univariate analysis identified age >65 years (HR 1.45, p = 0.015), tumor size >3 cm (HR 1.41, p = 0.018), non-skip N2 pattern (HR 1.58, p = 0.010), squamous histology (HR 1.36, p = 0.048), G3 grade (HR 1.72, p <0.001), lymphovascular invasion (HR 1.93, p <0.001), and lack of adjuvant chemotherapy (HR 1.61, p = 0.009) as adverse prognostic factors. In multivariate Cox regression analysis (**Table 2**), the novel subclassification system independently predicted OS: intermediate-risk skip N2 showed an adjusted HR of 1.82 (95% CI: 1.15–2.88, p = 0.021) and high-risk skip N2 showed an HR of 2.45 (95% CI: 1.42–4.23, p = 0.001) relative to low-risk skip N2. Non-skip N2 disease remained independently associated with poorer survival (HR 1.64, 95% CI: 1.14–2.36, p = 0.008).

**Table 2:** Multivariate cox regression analysis for overall survival

Variable	Hazard ratio	95% CI	p-Value
Age >65 years	1.38	1.02–1.87	0.038
Tumor size >3 cm	1.35	0.98–1.86	0.067
Non-skip N2	1.64	1.14–2.36	0.008
Intermediate-risk skip N2 vs low-risk skip N2	1.82	1.15–2.88	0.021
High-risk skip N2 vs low-risk skip N2	2.45	1.42–4.23	0.001
G3 grade	1.68	1.23–2.30	0.001
Lymphovascular invasion	1.87	1.36–2.57	<0.001

### Recurrence pattern analysis

Recurrence patterns differed substantially between groups (**Table 3**). The overall recurrence rate was lower in skip N2 patients (34.0% vs 45.4%, p = 0.045). When recurrence occurred, skip N2 patients demonstrated a higher proportion of distant-only metastases (58.8% vs 37.3% of recurrences, p = 0.024). The most common distant metastatic sites were brain (26.5%), bone (20.6%), and contralateral lung (17.6%). Local–regional recurrence as the first site of failure was less frequent in skip N2 patients (41.2% vs 62.7%, p = 0.021), suggesting enhanced local control efficacy in this subset.

**Table 3:** Recurrence patterns in skip N2 vs non-skip N2 patients

Recurrence type	Skip N2 (n = 17)	Non-skip N2 (n = 59)	p-Value
Local–regional only, n (%)	4 (23.5)	18 (30.5)	0.021
Distant only, n (%)	10 (58.8)	22 (37.3)	0.024
Both local and distant, n (%)	3 (17.6)	19 (32.2)	0.112
Total recurrence rate, n (%)	17 (34.0)	59 (45.4)	0.045

### Sensitivity analysis

To assess the robustness of our findings, we performed sensitivity analyses stratified by treatment period. In patients treated from 2015 to 2019 (n = 105), the prognostic advantage of skip N2 metastasis remained significant (HR 0.68, p = 0.034). Similarly, for patients treated from 2020 to 2022 (n = 75), the survival benefit persisted (HR 0.72, p = 0.044). The discriminatory ability of the novel subclassification system was maintained across both periods, with C-indices of 0.65 and 0.69, respectively. Exclusion of the 12 patients who received adjuvant immunotherapy did not materially alter the results, with the adjusted HR for high-risk skip N2 versus low-risk skip N2 remaining statistically significant at 2.38 (p = 0.002).

## Discussion

The present study proposes an anatomically grounded subclassification for skip N2 disease. We explicitly acknowledge that the proposed “direct” and “indirect” drainage patterns are inferred from anatomical surrogates rather than directly validated biological pathways; the biological interpretation of these patterns remains hypothetical. Our study employed the 8th edition TNM classification because our cohort spans from 2015 to 2022, predating widespread adoption of the N2a/N2b subclassification; our subclassification complements rather than competes with anatomical N2 staging. The present study demonstrates that skip N2 metastasis in resected NSCLC represents a biologically and prognostically distinct entity that can be further refined through integration of lymphatic drainage patterns and histological characteristics. Specifically, skip N2 metastasis occurred in 27.8% of pN2 patients and conferred a significant survival advantage over non-skip N2 disease (5-year OS 56.0% vs 41.5%, p = 0.028), consistent with prior reports of a 10%–15% absolute improvement in 5-year survival^4–6)^; lymphatic drainage patterns correlated with distinct clinicopathological features; and the composite subclassification successfully stratified pN2 patients into 3 risk groups with clinically meaningful survival differences (5-year OS: 71% vs 55% vs 27%, p = 0.001), outperforming conventional staging (C-index 0.67 vs 0.54, p = 0.008).

Our observation of superior survival in skip N2 patients (5-year OS 56.0% vs 41.5%) corroborates accumulating evidence challenging the traditional orderly progression model of lymphatic spread. This “skip N2 paradox” may reflect fundamental anatomical and clinicopathological differences rather than staging artifacts,^13)^ although the underlying biological mechanisms remain to be elucidated. The direct, indirect, and mixed drainage labels are anatomical hypotheses inferred from tumor location and nodal distribution, not biologically validated pathways. We cannot exclude that “direct” cases involved occult N1 micrometastases undetectable by conventional histopathology, nor confirm that “indirect” cases lacked transient direct lymphatic channels. These uncertainties limit definitive biological interpretation of drainage patterns from the present data. Nevertheless, the classification proved prognostically informative: direct drainage was associated with lower lymphovascular invasion than indirect pattern (23.8% vs 61.0%, p = 0.009), consistent with established evidence on LVI as an adverse prognostic factor in resected NSCLC,^14)^ while mixed pattern showed the most aggressive phenotype (5-year OS 27%).

Direct drainage tumors, typically peripheral and upper-lobe, exhibited low lymphovascular invasion and yielded a 71% 5-year overall survival rate, with a distant-only recurrence pattern consistent with adequate local control achieved by surgery.^12)^ Indirect drainage tumors, conversely, were centrally located, carried high rates of LVI and Grade-3 histology, and showed survival patterns more similar to conventionally advanced disease despite pathologically “negative” N1 nodes. Mixed-pattern cases combined both routes and displayed the most aggressive phenotype (27% 5-year survival). Multivariate analysis confirmed that LVI remained the strongest independent predictor of death (HR 1.87, 95 % CI 1.36–2.57, p <0.001),^12)^ while the proposed subclassification raised the concordance index to 0.67, significantly exceeding conventional pN2 staging (0.54, p = 0.008) and providing clinicians with a preliminary framework for risk stratification.

These observations offer a hypothesis-generating anatomical framework to reconcile the long-standing “skip N2 paradox”: so-called skip metastases are hypothesized to reflect either anatomical shortcuts with less metastatic burden or occult N1 micrometastases with more advanced disease biology.^13)^ These interpretations are based on anatomical and histological surrogates and remain hypothetical until validated by molecular studies. The composite model integrating drainage pattern, grade, histological subtype, and LVI raised the concordance index to 0.67, significantly exceeding conventional pN2 staging (0.54, p = 0.008). If validated in larger cohorts, this framework could inform the design of future prospective trials: high-risk skip N2 patients might represent a subgroup in whom the benefit of intensified perioperative strategies warrants evaluation, particularly given recent advances in adjuvant immunotherapy for resected stage III NSCLC.^15,16)^ While low-risk skip N2 patients should not be considered candidates for treatment de-escalation without prospective validation, their favorable prognosis may partly reflect the benefit of standard adjuvant therapy. It is critical to emphasize that the present study demonstrates prognostic stratification, not predictive utility for specific adjuvant therapies. Whether patients in different risk groups derive differential benefit from chemotherapy, immunotherapy, or radiotherapy remains unknown. These treatment implications are strictly hypothetical and should not guide clinical decision-making until validated by randomized studies.

It is important to emphasize that the proposed direct, indirect, and mixed drainage patterns are anatomical hypotheses inferred from tumor location and nodal distribution, not evidence of distinct biological pathways. Validation of these hypotheses requires future studies incorporating lymphangiogenesis markers (e.g., VEGF-C/D, podoplanin), epithelial–mesenchymal transition indicators (e.g., E-cadherin, vimentin), immune microenvironment profiling, and molecular nodal analysis. Real-time lymphatic mapping techniques such as indocyanine green near-infrared fluorescence imaging or sentinel node biopsy with ultrastaging could objectively classify drainage patterns and test whether the observed prognostic differences reflect true biological heterogeneity or anatomical variation alone.

Several limitations warrant consideration. First, lymphatic drainage routes were inferred from preoperative CT location and pathological nodal maps rather than prospectively validated tools such as lymphoscintigraphy or indocyanine green near-infrared imaging. By nature, this classification is inferential and should be regarded as hypothesis-generating rather than definitively mechanistic; the biological interpretation of “direct” versus “indirect” drainage proposed herein remains speculative and requires validation through prospective studies incorporating molecular nodal analysis and real-time lymphatic mapping. We emphasize that the proposed drainage patterns are anatomical hypotheses inferred from clinical data, rather than evidence of distinct biological pathways. Specifically, we cannot definitively exclude that some tumors classified as “direct drainage” may have traversed occult intrapulmonary nodal micrometastases undetectable by conventional histopathology, which would biologically reclassify them as indirect drainage; conversely, we cannot confirm that “indirect” cases did not involve transient direct lymphatic channels. This uncertainty directly affects the biological interpretation of drainage patterns and underscores the need for molecular nodal analysis in future validation studies. Second, the number of mixed-pattern cases was small (n = 6), limiting statistical power to detect interactions between drainage type and histological variables. The observed aggressive phenotype in this subgroup may therefore reflect chance variation rather than a true biological distinction, and should be interpreted with caution. Third, the proposed composite subclassification system was derived from a single-institutional retrospective cohort without external validation and should be regarded as an exploratory, hypothesis-generating framework; prospective, multi-institutional validation with standardized pathological review is essential before clinical application. While the score was constructed a priori, the limited sample size (n = 50 skip N2) precludes reliable assessment of model stability, and the C-index of 0.67 may be optimistically biased. Fourth, heterogeneity in chemotherapy regimens and the inclusion of 12 patients who received adjuvant immunotherapy may have influenced survival estimates; however, sensitivity analyses restricted to the pre-immunotherapy era (2015–2019) yielded identical hazard ratios. Only 34% of patients reached the 5-year assessment at the time of analysis (median follow-up 43 months). The reported 5-year survival rates are therefore Kaplan–Meier estimates with wide confidence intervals rather than observed proportions and may be optimistically biased if patients lost to follow-up had differential survival. Longer follow-up is needed to confirm these estimates and capture late recurrences beyond 5 years. Finally, the model was developed in only 50 skip N2 patients, including 11 high-risk cases, limiting precision of effect estimates and precluding reliable subgroup analyses. These findings should be regarded as exploratory and require validation in larger cohorts. Future validation efforts should ideally incorporate emerging lymphatic mapping techniques such as indocyanine green near-infrared fluorescence imaging or sentinel node biopsy with molecular nodal analysis, which may enable real-time, objective classification of drainage patterns and reduce the inferential uncertainty inherent in our retrospective approach, before the proposed classification is adopted to guide precision adjuvant strategies in resected NSCLC with mediastinal nodal involvement.

## Conclusions

Skip N2 metastasis in resected NSCLC represents a heterogeneous entity with distinct anatomical and clinicopathological features that can be stratified by inferred lymphatic drainage patterns and histological characteristics. Our novel 3-tiered subclassification system successfully stratifies pN2 patients into prognostically meaningful groups, outperforming conventional staging and providing a hypothesis-generating anatomical model that improves prognostic understanding of skip N2 disease. Direct drainage patterns were associated with a favorable prognosis and less aggressive histological features, while indirect and mixed patterns identified higher-risk patients. The biological mechanisms underlying these associations—whether anatomical shortcuts, occult N1 micrometastases, or alternative pathways—cannot be determined by the present inferential classification and require validation through prospective studies incorporating molecular nodal analysis (e.g., cytokeratin immunohistochemistry for occult micrometastases) and real-time lymphatic mapping (e.g., indocyanine green near-infrared fluorescence imaging). This integrated anatomical model offers a preliminary approach for precision oncology in NSCLC patients with mediastinal nodal involvement, pending external validation.

## Declarations

### Data availability

The experimental data used to support the findings of this study are available from the corresponding author upon reasonable request.
